# Is an improvement in anaemia and iron levels associated with the risk of early postpartum depression? A cohort study from Lagos, Nigeria

**DOI:** 10.1186/s12889-025-21942-x

**Published:** 2025-02-28

**Authors:** Ochuwa Adiketu Babah, Lenka Beňová, Elin C. Larsson, Claudia Hanson, Bosede Bukola Afolabi

**Affiliations:** 1 Department of Global Public Health, Karolinska Institutet, Stockholm, Sweden; 2 Department of Public Health, Institute of Tropical Medicine, Antwerp, Belgium; 3 Department of Obstetrics and Gynaecology, Faculty of Clinical Sciences, College of Medicine, University of Lagos, Lagos University Teaching Hospital, Idi-Araba, Lagos, Nigeria; 4 Department of Women’s and Children’s Health, Karolinska Institutet, Stockholm, Sweden; 5 Centre for Clinical Trials, Research and Implementation Science, College of Medicine, University of Lagos, Idi-Araba, Lagos, Nigeria

**Keywords:** Anaemia, Haemoglobin concentration, Improvement, Iron deficiency, Mental health, Perinatal mental health, Nigeria, Sub-saharan Africa, maternal mortality, maternal morbidity, Postpartum depression, Postpartum haemorrhage, Pregnancy, Severity

## Abstract

**Background:**

Anaemia and depression are common conditions which affect pregnant and postpartum women. Evidence points to associations between anaemia and iron deficiency during pregnancy, and mental health disorders like depression. However, it is unclear the association between improvement in anaemia severity or iron levels during pregnancy and incidence of postpartum depression.

**Objectives:**

This study examined association between improvement in anaemia severity and iron levels during pregnancy after four weeks of treatment and the incidence of depression at two weeks postpartum.

**Methods:**

This cohort study nested within a clinical trial in Lagos Nigeria, included 438 anaemic (haemoglobin concentration < 11 g/dL) pregnant women at 20–32 weeks’ gestation without depression followed up until two weeks postpartum. Participants received either intravenous or oral iron treatment at enrolment. Repeat screening for anaemia and iron deficiency (serum ferritin < 30ng/mL) was done at four weeks post-treatment. The outcome, depression (score > 10), was measured at two weeks postpartum using validated Edinburgh Postnatal Depression Scale. Associations between improvement in anaemia severity and iron levels after four weeks post-enrolment versus depression at two weeks postpartum were examined using logistic regression analysis, adjusting for confounders.

**Results:**

Mean age of women was 29.5 ± 5.6years. Median haemoglobin concentration of 9.3 (IQR: 8.8–9.8)g/dL and median serum ferritin 44.4 (IQR: 22.1–73.7)ng/mL at enrolment. Prevalence of postpartum depression was 5.8% (95%CI: 3.8–8.5%). There was a non-significant association between improvement in anaemia severity at four weeks post-enrolment and postpartum depression, aOR: 0.15 (95%CI: 0.02–1.15). The odds for postpartum depression was nearly five times higher in women who had postpartum haemorrhage, aOR: 4.90 (95%CI: 1.18–20.36). In the subgroup with iron deficiency (*n* = 148), no association was found between an improvement in iron levels four weeks post-enrolment and the odds for postpartum depression, aOR: 1.14 (95%CI: 0.09–3.93).

**Conclusion:**

Improvement in anaemia severity during late pregnancy was non-significantly associated with lower risk for postpartum depression; no association between improvement in iron levels and postpartum depression. It is likely that an improvement in anaemia severity in early pregnancy will lessen the burden of postpartum depression; however, this study is limited by sample size to draw this conclusion.

**Supplementary Information:**

The online version contains supplementary material available at 10.1186/s12889-025-21942-x.

## Introduction

Anaemia, a non-communicable disease of public health concern, affects 32 million pregnant women globally every year [1]. Anaemia has adverse consequences on maternal and foetal health including a higher rates of preterm birth, miscarriage, low birth weight, and perinatal death [2]. Recent evidence points to associations between anaemia/ iron deficiency in pregnancy, and the risk for postpartum depression [3–7]. Depression is a neglected non-communicable disease affecting 280 million people worldwide, with 0.7 million cases ending as suicidal deaths yearly [8]. Globally, depression affects 10% of women during pregnancy and 13% postpartum [9]. The burden of both maternal anaemia and postpartum depression is higher in low-middle-income countries (LMICs) compared to high-income countries (HICs) [1, 10]. The prevalence of postpartum depression is estimated to be 10% in HICs and 19% in LMICs; [11] with a pooled prevalence of 19% in sub-Saharan Africa [12]. The prevalence of anaemia in pregnancy was reported to be 17% in HIC and 46% in LMICs including sub-Saharan Africa in 2019 [13]. The reasons for these differences are multifactorial, and include the higher rates of poverty, malnutrition, lower literacy rate, and violence of various forms like intimate partner violence in LMICs compared to HICs [10, 11, 14]. 

Depression during pregnancy is associated with 1.40 times higher risk of preterm birth and 1.49 times higher risk of low birth weight babies [15]. Risk factors for postpartum depression include various forms of violence such as intimate partner violence, lack of partner or societal support, socioeconomic status, birth experience and a history of psychiatric illness including pre-existing depression [16]. Evidence from a recent meta-analysis points to an increased risk of having symptomatic postpartum depression in the presence of postpartum anaemia or iron deficiency [17]. 

Anaemia causes a lack of oxygen supply to the brain affecting its function. Anaemia has been found to cause impaired myelination and monoamine metabolism in the brain with resultant reduction in the levels of neurotransmitters like serotonin, dopamine, and norepinephrine [6, 18]. This in turn alters the emotional and psychological function of affected individuals predisposing to psychiatric disorders like depression [6]. In addition, the symptoms of anaemia such as weakness, fatigue and difficulty in breathing with resultant lack of interest in daily activities can predispose to depression.

Most studies on the association between anaemia and postpartum depression were conducted in HIC and found anaemia in the antepartum and postpartum periods to be associated with an increased risk of postpartum depression [19]. These studies were cross-sectional and examined haemoglobin (Hb) concentration at one time point during pregnancy [19]. Considering the disparities between HICs and LMICs in terms of nutrition, culture, lifestyle, socioeconomic status and healthcare infrastructure, and thus potential difference in risk for depression, there is a need to conduct studies evaluating the association between anaemia and postpartum depression in sub-Saharan Africa. In addition, the impact of an improvement in anaemia severity or iron levels over time during pregnancy on postpartum depression is poorly understood. Evidence from a recent study on adults aged 65 years and above in Quebec City of Canada found that the odds for depression is more than doubled when anaemia is untreated; with anaemia treatment the risk for depression is similar to that among persons without anaemia [6]. This suggests that an improvement in levels of Hb concentration over time might have an effect on the risk of developing depression. It would be important to evaluate this association in pregnant women considering the effect of pregnancy on the haematological system, and the high burden of the two conditions.

Nigeria has a high burden of anaemia in pregnancy and postpartum depression with a prevalence of 61% and 36%, respectively [20, 21]. Among pregnant women with moderate to severe anaemia, 41% are estimated to have iron deficiency [22]. Despite the high burden, anaemia in pregnancy and in particular postpartum depression are under-researched in Nigeria. Earlier studies from Nigeria have focused on prevalence and risk factors for maternal anaemia or postpartum depression separately [21–25]. 

There is a paucity of studies evaluating the association between maternal anaemia and postpartum depression in sub-Saharan Africa probably due to poor funding of research, non-inclusion of depression screening in routine antenatal and postpartum care, and oversight of the disease burden. The current study, we believe will promote inclusivity from global health perspective and provide further evidence on the association between anaemia and postpartum depression and the significance of prompt anaemia treatment during pregnancy. We examined the association between improvement in the severity of anaemia after four weeks of treatment in late second and early third trimester of pregnancy (and separately, improvement in iron levels) and the incidence of depression at two weeks postpartum.

## Methods

### Study design

This prospective cohort study was nested within the IVON trial a hybrid type 1 study combining a randomised controlled trial and an implementation science study [26]. IVON compared the effectiveness and safety of intravenous ferric carboxymaltose and oral ferrous sulphate for treating iron deficiency anaemia during pregnancy. To provide valuable evidence for the facilitation of the implementation of the use of intravenous ferric carboxymaltose for anaemia treatment during pregnancy in Nigeria and other LMICs, the IVON trial compared the prevalence of important clinical outcomes (maternal and foetal) including postpartum depression in women who had their anaemia treated with intravenous versus oral iron. It also explored the acceptability, feasibility, and fidelity of the use of intravenous iron during pregnancy for iron deficiency anaemia treatment in Nigeria and currently analysing data on the cost-effectiveness of the drugs. The enrolment period spanned between August 9, 2021 and December 15, 2022 [27]. 

### Study setting

We included the data from all five health facilities in Lagos state which were part of the IVON trial. All five were public facilities: one tertiary hospital, two secondary health facilities, and two primary health centres. These facilities were purposively selected because of their proximity to one another to facilitate effective coordination of trial research and referrals.

### Participants

This study included 438 pregnant women, aged 15–49 years and at gestational ages of 20–32 weeks who had anaemia (Hb concentration < 11 g/dL) as detected from their Hb concentration from an autoanalyzer. Excluded from the IVON trial were women who had haemoglobinopathies such as sickle cell disease and thalassemia, recent bleeding, blood transfusion in the last three months, human immunodeficiency virus infection, severe asthma, immune-related diseases like systemic lupus erythematosus or rheumatoid arthritis, and those who had a history of allergy to iron. In addition, for this study, we excluded women who met criteria for depression on screening with Edinburg Postnatal Depression Scale (EPDS) > 10 at enrolment and those with multiple gestations. All the women received iron supplements for anaemia treatment either as oral iron (ferrous sulphate) or as intravenous iron (ferric carboxymaltose).

### Sample size calculation

A sample size of 603 pregnant women was found to be sufficient for this study using the formula for cohort study sample size calculation by Sharma et al. [28], with a statistical power of 80% at 95% confidence level, considering an incidence rate for postpartum depression of 18.9% in pregnant women with anaemia and 12.8% in pregnant women without anaemia as found in a previous study in conducted China, as there was no suitable local study for sample size calculation [29], adjusting for 20% attrition. However, all eligible pregnant women who participated in the IVON trial in Lagos State were included in this study.

### Data collection

All women at gestational ages of 20–32 weeks receiving antenatal care at the study sites had point-of-care testing for anaemia with a Hemocue 301 device (Danaher corporation, Brea, California, U.S.A.) as part of their routine antenatal care. Those found to be moderately or severely anaemic (Hb concentration < 10 g/dL) were invited to participate in the IVON trial [27]. Potential participants were informed about the study in details and given an opportunity to ask questions. Those who consented were further screened for eligibility. Eligible women had their blood specimen collected for complete blood count assay using autoanalyzer (SYSMEX XN-L 500 SERIES, Japan), and for serum ferritin assay using ARCHITECT (by ABBOTT). Only participants who were confirmed to have anaemia (Hb concentration < 11 g/dL) based on the complete blood count result and received treatment were included in this analysis. We preferred to use the autoanalyzer Hb concentration of the participants for this study because the autoanalyzer which is considered the goal standard for determination of haemoglobin concentration has been found to be more accurate compared to the Hemocue; capillary blood Hemocue has been found to be associated with an overestimation of anaemia [30, 31]. 

**Fig. 1:**
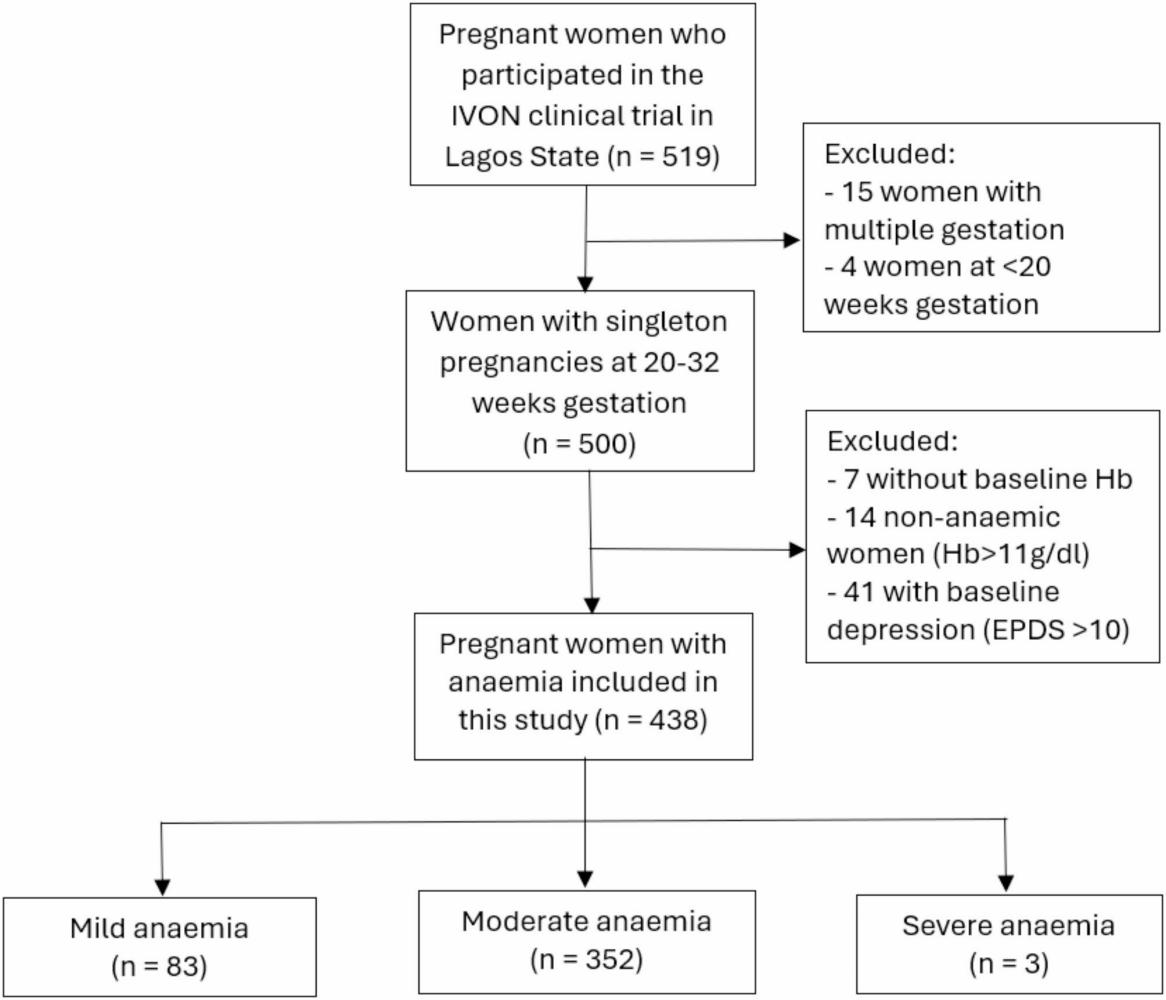
Flow chart on participants’ inclusion in this study

In addition, sociodemographic and obstetric data were collected at enrolment onto REDCap by the research nurses. These included age, level of education, place of residence, income, parity, and date of last menstrual period. Gestational age was calculated from the last menstrual period if a woman was sure of her date or from an early scan done before 22 weeks gestational age if she was unsure of her date. Figure 1 is a flow chart summarising inclusion of participants in this study.

### Follow-up

**Fig. 2:**
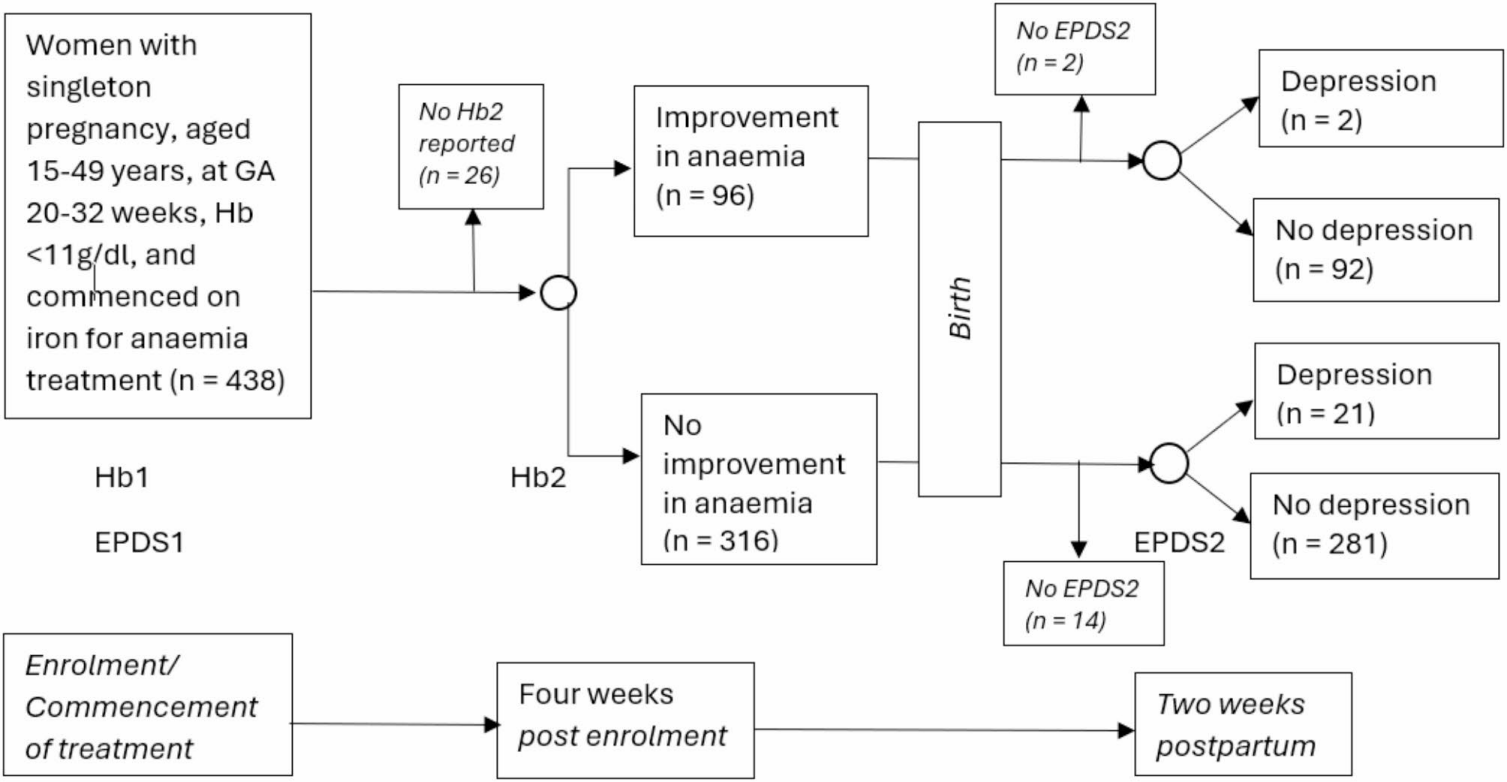
Cohort study flow diagram. Hb- haemoglobin concentration measured in grammes per decilitre. Hb1- enrolment, Hb2- four weeks post-enrolment. Improvement in anaemia severity refers to a reduction in the severity of anaemia, on the basis of Hb1 vs. Hb2. EPDS - Edinburg Postnatal Depression Scale used in assessing for probable depression. EPDS1 -score at enrolment, EPDS2 -four weeks post-enrolment

For this study, the follow-up period comprised three visits post-enrolment, that is, at four weeks post-enrolment, at delivery and at two weeks postpartum. At four weeks post-enrolment, the women had a repeat complete blood count and serum ferritin assay. At the time of childbirth, data were collected for pregnancy complications such as preterm delivery, hypertensive disorders, gestational diabetes, occurrence of postpartum haemorrhage and foetal survival (dead or alive) [26]. At two weeks postpartum, women were again screened for depression. Figure 2 shows the flow diagram for the cohort.

### Exposure

The exposures of interest were improvement in anaemia severity and improvement in iron levels after four weeks post-enrolment. Anaemia in pregnancy was defined as a Hb concentration < 11 g/dL [32]. The severity of anaemia was categorized as mild if Hb concentration is 10.0–10.9 g/dL, moderate if 7.0–9.9 g/dL and severe if < 7.0 g/dL [32]. Iron levels were defined using a serum ferritin cut-off value of 30 ng/mL and categorized as iron deficient (< 30 ng/mL) or not iron deficient (iron replete) if serum ferritin was ≥ 30 ng/mL.

Improvement in the level of both exposures was defined by the direction of change in levels. Improvement in anaemia severity was categorized as improved if the severity improved after four weeks post-enrolment, which is: from severe to moderate, severe to mild, severe to no anaemia, moderate to mild, moderate to no anaemia, or mild to no anaemia. It was categorized as unimproved if the severity of anaemia remained the same or worsened by a category at four weeks’ post-enrolment. On the other hand, improvement in iron levels from enrolment to four weeks post-enrolment was categorized as resolved or persistent. Those whose iron levels changed from being iron deficient at enrolment to iron replete after four weeks post-enrolment were categorized as resolved. Those who were iron deficient at enrolment and remained iron deficient after four weeks post-enrolment were categorized as persistent.

### Outcome

The outcome of interest was occurrence of depression at two weeks postpartum defined as an EPDS score > 10 [33]. The EPDS is a validated 10-item questionnaire, which has an internal consistency of 0.83 and an area under the receiver operation characteristic curve of 0.83 [34]. The English version, which was used in this study, was previously validated in Nigeria and found to be a good screening tool for minor and major depression in late pregnancy [35]. A recent meta-analysis found a cut-off of > 10 for diagnosing depression in pregnant and postpartum women to have the best-combined sensitivity and specificity compared to other cut-off values such as > 9 or > 13.^33^

### Data analysis

Data were analyzed using Stata version 18.0 (StataCorp. 2023. *Stata Statistical Software: Release 18*. College Station, TX: StataCorp LLC). Descriptive statistics were done. Normally distributed continuous variables were presented as mean and standard deviation while those skewed were presented as median and interquartile range. Normality testing was done using Shapiro Wilks test. Categorical variables were presented as frequency and percentage. Prevalence of depression at two weeks postpartum were presented as proportion with 95% confidence intervals. Associations between the severity of anaemia at enrolment and depression at two weeks postpartum on one hand, and between categories of iron levels at enrolment versus depression at two weeks postpartum on the other hand, were determined using Chi square test.

Logistic regression analysis was used to determine the association between improvement in anaemia severity from enrolment to two weeks postpartum and depression at two weeks postpartum. Purposeful selection was used for multivariable logistic regression analysis using a 3-step approach [36]. Step 1, bivariate logistic regression with p-value threshold of 0.25 for variables selection (age and body mass index dropped). Exact logistic regression analysis was used for variables with an empty cell in the 2 × 2 tables. Step 2, multivariable logistic regression analysis on variables selected in step 1 and p-value threshold of 0.05 for retention of variables in the model. Step 3, variables retained in step 2 (postpartum haemorrhage) including the predictor of interest (improvement in anaemia severity) were included in the model. Subsequently, variables dropped in steps 1 and 2 were included in sequence but none met the criteria for inclusion into the model based on a change in beta coefficient of the other variables by ± 10%. Next, potential confounders of depression like sociodemographic variables (age and income) and stressors (adverse foetal outcomes, postpartum haemorrhage, and other pregnancy complications) were adjusted for in the final model. Postpartum haemorrhage was considered separately because of its direct association with anaemia while all other complications occurring during pregnancy like hypertensive disorders, gestational diabetes, cardiomyopathy, preterm birth, etc. were categorized together. The same approach was used to determine the association between improvement in iron levels at four weeks post-enrolment versus risk of depression at two weeks postpartum among the subgroup of the anaemic pregnant women who had iron deficiency at enrolment. Crude and adjusted odds ratios for postpartum depression were presented.

In addition, an exploratory analysis was done to determine the correlation between change in haemoglobin concentration from enrolment to four weeks post-commencement of treatment and EPDS score at two weeks postpartum using Spearman’s rank correlation coefficient, and the association between these two variables was further explored using simple linear regression analysis. The relationship between the two variables were summarised on a two-way graph applying spline to depict non-linear association. We also determined if any difference in the incidence of depression (using Chi Square test) and median EPDS scores (using Wilcoxon ranksum test) at two weeks postpartum in women who had anaemia treated during pregnancy with intravenous ferric carboxymaltose versus oral ferrous sulphate. We presented the results on a bar chart and a box plot.

Statistical significance was set at an alpha level of 5% assuming a two-tailed hypothesis.

### Missing data

Of the 438 women included in this study, 26 lacked Hb2 measure and of these, nine did not have EPDS2 as well; 16 did not have EPDS2 measures only (Fig. 2). In addition, there were eight missing values for gestational age at delivery; 25 missing values for income; eight for pregnancy complications; 38 for estimated blood loss at delivery; 12 for mode of delivery; and nine for foetal outcomes. Imputation was not done for missing values during data analysis.

### Ethics

Ethical approvals were obtained for the IVON trial from institutional ethics committees - Lagos University Teaching Hospital HREC (ADM/DCST/HREC/APP/3971), State Ministry of Health, Kano (MOH/Off/797/T.1/2102), Aminu Kano Teaching Hospital, Kano (NHREC/28/01/2020/AKTH/EC/2955), and the National Health Research Ethics Committee (NHREC/01/01/2007-04/02/2021).^26^ In addition, permission to conduct research in the secondary and primary health facilities in Lagos state was officially sought from Lagos State Health Service Commissions (LSHSC/2222/VOLIII), and Lagos state Primary Health Care Board (LS/PHCB/MS/1128/VOL.VII/100) respectively. The participants were given full autonomy to decide on their participation in the research and were informed of their right to withdraw at any stage. They all signed informed consent prior to data collection at the health facilities. All participants enjoyed equal rights like other hospital patients. To maintain confidentiality, the data released for this study was de-identified. Participants who screened positive for depression at either enrolment or postpartum follow-up were referred to the collaborating psychiatrist for further evaluation and treatment at no cost.

## Results

Of the 438 pregnant women included in this study, the mean age at enrolment was 29.5 ± 5.6 years. Table 1 shows a summary of the participants’ demographic and clinical characteristics. Median Hb concentration was 9.3 (IQR: 8.8–9.8, range 6.5–10.9) g/dL at enrolment and 9.7 (IQR: 9.2–10.2, range 5.0–12.7) g/dL at four weeks post enrolment. Their median serum ferritin level was 44.3 (IQR: 22.1–73.7) ng/mL at enrolment and 144.8 (IQR: 52.5–305.9) ng/mL after four weeks post-enrolment. Of the 438 anaemic pregnant women at enrolment, 285 (65.1%) were iron replete while 153 (34.9%) were iron deficient. The incidence of postpartum depression was 5.8% (95%CI: 3.8-8.5%).

**Table 1:** Sociodemographic characteristics and clinical profile of study participants (*n* = 438)

Variables	Mean (S.D.)
Maternal age (years)	29.5 ± 5.6
	**Median (IQR)**
Haemoglobin concentration at enrolment (g/dL)	9.3 (8.8–9.8)
Haemoglobin concentration at four weeks post-enrolment (g/dL), *n* = 412	9.7 (9.2–10.2)
Serum ferritin at enrolment (ng/mL)	44.3 (22.1–73.7)
Serum ferritin at four weeks post-enrolment (ng/mL), *n* = 414	144.8 (52.5–305.9)
Gestational age at enrolment (weeks)	24 (22–28)
Gestational age at delivery (weeks), *n* = 430	39 (38–40)
	**Frequency (%)**
Iron status at enrolment	
Iron deficient	153 (34.9%)
Iron replete	285 (65.1%)
Trimester at enrolment	
Late second trimester (20–26 weeks)	275 (63.8%)
Early third trimester (27–32 weeks)	163 (37.2%)
Place of residence	
Urban	385 (87.9%)
Rural	53 (12.1%)
Highest level of education^¶^	
No formal education	4 (0.9%)
Primary	15 (3.4%)
Secondary	198 (45.2%)
Tertiary	221 (50.5%)
Participant’s monthly income (*n* = 413)*	
<₦50,000	219 (53.0%)
<₦50,000 – ₦100,000	121 (29.3%)
>₦100,000	73 (17.7%)
Body mass index at enrolment	
Underweight (< 18.5 kg/m2)	3 (0.7%)
Normal (18.5 - <25 kg/m2)	198 (45.2%)
Overweight (25 - <30 kg/m2)	148 (33.8%)
Obese (30 kg/m2)	89 (20.3%)
	**Proportion (95%CI)**
Depression at two weeks postpartum (*n* = 413)	5.8% (3.8–8.5%)
Pregnancy complications (*n* = 430)^#^	6.3% (4.2–9.0%)
Postpartum haemorrhage (*n* = 398)	4.5% (2.7–7.1%)
Adverse fetal outcomes e.g., stillbirths, prematurity, foetal anomaly, neonatal unit admission (*n* = 429)	4.0% (2.3–6.3%)

^¶^Highest level of education - highest attained whether or not it was completed. ₦ – Nigerian Naira; *₦448 ≡ $1 as of 15 December 2022 when the last participant entered IVON trial. S.D. – Standard deviation; n – total counts; n – denominator each indicator; % – percentage; 95%CI – 95% confidence interval. ^#^Pregnancy complications -any condition during pregnancy that increases the risk of morbidity or mortality to the mother and/or baby

Figure SM1 is a Sankey diagram showing the pattern of improvement in the severity of anaemia from enrolment to four weeks post-enrolment among the study participants. Of the 438 at enrolment, 83 (18.9%) had mild anaemia, 352 (80.4%) had moderate anaemia and three (0.7%) had severe anaemia. Of the participants enrolled, only 412 had Hb concentration reported at four weeks post enrolment. Among those who had mild anaemia at enrolment (*n* = 83), 17 (20.5%) achieved anaemia correction following four weeks of treatment, 52 (62.7%) remained mildly anaemic, nine (10.8%) worsened to moderate severity and one (1.2%) worsened to severe anaemia. Among those who had moderate anaemia at enrolment (*n* = 352), 10 (2.8%) achieved correction in four weeks, 67 (19.0%) improved to mild anaemia, 252 (71.6%) remained moderately anaemic while one (0.3%) developed severe anaemia. Among those with severe anaemia at enrolment (*n* = 3), two (66.7%) improved to moderate anaemia while one (33.3%) remained severely anaemic after four weeks of treatment, Figure SM2 shows improvement in iron levels. Of those iron deficient (*n* = 153), 119 (77.8%) became iron-replete four weeks post-enrolment while 25 (16.3%) had persistent iron deficiency. Of those who were iron replete at baseline, 12 (4.2%) became iron deficient at four weeks post-enrolment. Figure SM3 shows that 96 of 412 (23.3%) participants improved in anaemia severity at four weeks post-enrolment and this did not differ by route of iron supplementation (*p* = 0.191).

Table 2 shows the distribution between different categories of anaemia severity or iron deficiency at enrolment and four weeks postpartum versus the prevalence of depression at two weeks postpartum. In this crude analysis using Chi-square test, there was no statistically significant association observed between the severity of anaemia at enrolment (*p* = 0.624) and the prevalence of depression at two weeks postpartum. There was also no statistically significant association between iron deficiency at enrolment and depression at two weeks postpartum (*p* = 0.792).

Next, we present the bivariate (crude) and multivariable (adjusted) logistic regression model results examining associations between improvement in anaemia severity (Table 3) and categories of improvement in iron levels (Table 4) during pregnancy and risk for depression at two weeks postpartum. We found a non-significant association between improvement in anaemia severity and postpartum depression in the crude model OR = 0.29 (95%CI: 0.07–1.26). The direction of the association remained the same and the magnitude of the effect increased after adjusting for confounders aOR = 0.15 (95%CI: 0.02–1.15) and remained marginal (*p* = 0.068). Women who had postpartum haemorrhage had 4.9 times greater adjusted odds of having postpartum depression compared to women who did not have postpartum haemorrhage, (95%CI 1.18–20.36), Table 3. There was no statistically significant association between improvement in iron levels and the odds of depression at two weeks postpartum among the subgroup of anaemic pregnant women who had iron deficiency at enrolment (*p* = 0.920), Table 4.

**Table 2:** Bivariate associations between severity of anaemia and iron levels versus postpartum depression

Status	Probable depression at two weeks postpartum		Chi-square *p*-value
	Yes	No	
ANAEMIA SEVERITY	*n* (%)	*n* (%)	
**At enrolment (n = 413)**	*n* = 24	*n* = 389	
Mild	3 (12.5%)	76 (19.5%)	0.624
Moderate	21 (87.5%)	310 (79.7%)	
Severe	0 (0.0%)	3 (0.8%)	
**At 4 weeks post-enrolment (n = 396)**	*n* = 23	*n* = 373	
No anaemia	0 (0.0%)	27 (7.2%)	0.374
Mild	5 (21.7%)	111 (29.8%)	
Moderate	18 (78.3%)	233 (62.5%)	
Severe	0 (0.0%)	2 ( 0.5%)	
**IRON LEVEL**	**n (%)**	**n (%)**	
**At enrolment (n = 413)**	*n* = 24	*n* = 389	
Iron deficient	8 (33.3%)	140 (36.0%)	0.792
Iron replete	16 (66.7)	249 (64.0%)	
**At 4 weeks post-enrolment (n = 397)**	*n* = 23	*n* = 374	
Iron deficient	22 (95.7%)	35 (9.4%)	0.417
Iron replete	1 (4.3%)	339 (90.6%)	

Percentages presented are of column total. Missingness – 25 women did not have an EPDS score at two weeks postpartum. Cut-off values: No anaemia - Hb ≥ 11.0 g/dL; Mild anaemia - Hb 10.0–10.9 g/dL; Moderate anaemia - Hb 7.0-9.9/dL; Severe - Hb < 7.0 g/dL; Iron deficient - Serum ferritin < 30ng/mL; Iron replete - Serum ferritin ≥ 30ng/mL

**Table 3:** Associations between improvement in anaemia severity during pregnancy and risk for postpartum depression

Predictors	Bivariate analysis			Multivariable analysis (*n* = 351)	
	*N*	OR (95%CI)	*p*-value	aOR (95%CI)	*p*-value
Improvement in anaemia severity	396				
Improved	94	0.29 (0.03–1.23)	0.118	0.15 (0.02–1.15)	0.068
Unimproved	302	Ref	-	Ref	-
Maternal age*	**413**	0.97 (0.90–1.04)	0.378	0.98 (0.90–1.07)	0.621
Gestational age at enrolment*	**413**	0.94 (0.83–1.07)	0.352	0.93 (0.80–1.07)	0.311
**Place of residence**	**413**				
Rural	49	0.21 (0.00–1.20)	0.088		
Urban	364	Ref			
**Income**	**390**				
<₦50,000	206	Ref	-	Ref	-
<₦51,000 – ₦100,000	113	1.35 (0.53–3.46)	0.531	1.17 (0.38–3.67)	0.771
>₦100,000	71	1.34 (0.45–4.01)	0.597	1.66 (0.52–5.28)	0.394
**Pregnancy complications**	**413**				
Yes	27	1.32 (0.29–5.95)	0.715	1.43 (0.28–7.24)	0.669
No	386	Ref	-	Ref	-
**Postpartum haemorrhage**	**385**				
Yes	18	3.88 (1.03–14.62)	0.045	4.90 (1.18–20.36)	**0.029**
No	367	Ref	-	Ref	-
**Adverse foetal outcome**	**413**				
Yes	17	1.08 (0.14–8.55)	0.941	2.26 (0.22–23.18)	0.493
No	396	Ref	-		

Likelihood ratio p-value for multivariable model = 0.156, Hosmer and Lemeshow goodness of fit p-value = 0.314, Tolerance = 0.97, VIF = 1.03

*Inputted in the model as continuous variables

Postpartum haemorrhage refers to blood loss above 500mL in women who had vaginal delivery and above 1000mL in women who had caesarean section. The multivariable model did not converge when place of residence was added

Pregnancy complications refer to any condition occurring during pregnancy that increases the risk of morbidity or mortality to the mother and/or her baby, like preeclampsia/ eclampsia, gestational hypertension, gestational diabetes, peripartum cardiomyopathy, etc. but excludes postpartum haemorrhage

**Table 4:** Association between improvement in iron levels and risk for postpartum depression

Predictors	Bivariate analysis			Multivariable analysis (*n* = 116)	
	*N*	OR (95%CI)	*p*-value	aOR (95%CI)	*p*-value
Improvement in iron levels	139				
Improved (Resolved)	115	1.49 (0.17–12.71)	0.715	1.14 (0.09–13.93)	0.920
Unimproved (Persistent)	24	Ref	-	Ref	-
Maternal age*	**148**	0.92 (0.80–1.06)	0.226	0.92 (0.77–1.11)	0.373
Gestational age at enrolment*	**148**	1.03 (0.84–1.26)	0.762	1.14 (0.85–1.52)	0.398
**Place of residence**	**148**				
Rural	21	0.53 (0.00–3.60)	0.569		
Urban	127	Ref			
**Income**	**139**				
<₦50,000	74	Ref	-	Ref	-
<₦51,000 – ₦100,000	42	1.82 (0.35–9.45)	0.476	4.64 (0.38–56.56)	0.229
>₦100,000	23	2.25 (0.35–14.40)	0.390	7.02 (0.54–91.84)	0.137
**Pregnancy complications**	**148**				
Yes	10	1.22 (0.00–8.88)	> 0.999		
No	138	Ref	-		
**Postpartum haemorrhage**	**132**				
Yes	9	3.72 (0.37–37.29)	0.264	2.78 (0.23–33.54)	0.420
No	123	Ref	-	Ref	-
**Adverse foetal outcome**	**148**				
Yes	6	2.14 (0.00–16.88)	> 0.999		
No	142	Ref	-		

Analysis included subgroup of pregnant women with iron deficiency anaemia at enrolment only

Likelihood ratio p-value for multivariable model = 0.540, Hosmer and Lemeshow goodness of fit p-value = 0.871, Tolerance = 0.96, VIF = 1.05

*Inputted in the model as continuous variables

Postpartum haemorrhage refers to blood loss above 500mL in women who had vaginal delivery and above 1000mL in women who had caesarean section. The multivariable model did not converge when place of residence, pregnancy complication and adverse foetal outcome were added

Pregnancy complications refer to any condition occurring during pregnancy that increases the risk of morbidity or mortality to the mother and/or her baby, like preeclampsia/ eclampsia, gestational hypertension, gestational diabetes, peripartum cardiomyopathy, etc

To explore further the association between change in haemoglobin from enrolment to four weeks post-commencement of treatment and EPDS score at two weeks postpartum, we assessed linear relationship as shown in Figure SM4. We did not find a significant association between the two variables, *r* = -0.019, *p* = 0.78. The spline line shows a non-linear association between the two variables. The equation obtained on simple linear regression analysis was y = 4.91–0.1x where: y = EPDS score at two weeks postpartum, and x is the change in haemoglobin concentration from enrolment to four weeks post commencement of treatment.

In Figure SM5, we explored the association between the type of drug used for anaemia treatment and the occurrence of depression at two weeks postpartum. There was no significant difference in the prevalence of postpartum depression in women who had intravenous versus oral iron for anaemia treatment, 7.1% vs. 4.5% respectively, *p* = 0.249 (Figure SM5A); there was also no significant difference in their median EPDS scores, 5 (IQR: 3–7) vs. 5 (IQR: 2–7) respectively (Figure SM5B).

## Discussion

This study examined the association between an improvement in anaemia severity and iron levels during pregnancy and depression at two weeks postpartum among women seeking care in five public health facilities in Lagos, Nigeria. It found a prevalence of 5.8% for postpartum depression among women who were diagnosed and treated for anaemia in the late second or early third trimesters of pregnancy. We found a non-significant association between an improvement in anaemia severity at four weeks post-enrolment and lower risk of postpartum depression at two weeks postpartum. There was no statistically significant association between improvement in iron deficiency and the risk of developing postpartum depression at two weeks post-delivery.

The prevalence of postpartum depression in this study was low compared to findings in previous studies in Nigeria and other countries, likely because we excluded women with depression at enrolment. Two studies in South-West Nigeria, reported a postpartum depression prevalence of 8.8% in Ekiti State and 35.6% in Lagos State [21, 37]. Like our study, these two studies assessed depression with the EPDS tool, but did not target anaemic women, and they assessed depression at six weeks postpartum. Our study included carefully selected trial subjects who received standard treatment for anaemia with high drug adherence rate of 94% among those who had oral iron [27]. 

A systematic review proposed that pooled prevalence for postpartum depression is 19% for sub-Saharan Africa [12]. The prevalence of postpartum depression varies widely across regions worldwide; highest prevalence of 61% in Afghanistan and lowest prevalence of 6.5% in Denmark in a study which mapped the global prevalence of postpartum depression from data of 565 studies across 80 countries [38]. The differences in prevalence rates across regions maybe due to societal differences, type of screening tool used and the study size [38]. In addition, the differences in prevalence across regions may be due to differences in participants’ characteristics, the type of screening tool used in assessing depression, and the cut-off values applied in defining depression. A few validation studies in different states in Nigeria have found lower thresholds for diagnosing postpartum depression with best cut off point on EPDS ranging from 7 to 9 [39, 40]. The use of a higher cut off point of > 10 in our study to identify women with postpartum depression could have contributed to under-reporting.

Association between anaemia or iron deficiency and the risk of postpartum depression had been found in previous studies [7, 41, 42]. We found a non-significant association between an improvement in anaemia severity and the risk of postpartum depression, and refuted the presence of an association between an improvement in iron levels and the risk of postpartum depression. However, in view of the large effect size, an 85% reduction in the odds of postpartum depression in pregnant women whose severity of anaemia improved four weeks after treatment, with very wide confidence interval, the result points to an inadequate power to detect the difference in effect among our sample.

In addition, the lack of certainty could also be due to the fact that the trial participants were all treated for anaemia; it is possible that this made their risk for postpartum depression approach that of non-anaemic pregnant women. This explanation is supported by a recent study which evaluated the effect of treating anaemia on the risk of postpartum depression and found that anaemia only increases the risk for depression if untreated, but if treated, the risk is similar to that of non-anaemic women [6] However, in our study not many of the pregnant women improved in anaemia treatment by four weeks following treatment. This is likely because only one-third of the participants had iron deficiency, Furthermore, the peak period of physiological haemodilution in pregnancy − 34–36 weeks gestation - falls within the four-week post-enrolment period making the impact of anaemia treatment less evident [43]. 

Despite a small sample size (*n* = 116), we observed that the direction of the effect of an improvement in iron status on the odds for postpartum depression was opposite to that observed for an improvement in anaemia severity. Improvement in iron status was associated with an increase in odds for postpartum depression. A review found that though several studies have reported iron deficiency to be negatively associated with postpartum depression, a few other studies have found that the risk for postpartum depression is increased in iron overload [44]. The possibility of asymptomatic iron overload cannot be ruled out in this study because half of the participants in the IVON TRIAL received loading dose of intravenous iron for anaemia treatment. These findings call for further research to determine the concentrations of serum iron that will promote an optimal mental health and the effect of iron metabolism on the pathogenesis of postpartum depression.

We found a strong association between postpartum haemorrhage and postpartum depression. This is corroborated by similar findings in a meta-analysis [45]. It is difficult to say if anaemia, a consequence of postpartum haemorrhage, is responsible for the increased odds of postpartum depression observed in this study. However, other factors like change in mood, lack of social support, stress and uncertainty surrounding delivery events might aggravate the risk of postpartum depression [46]. These we adjusted for within limitations of available data by including other pregnancy complications, occurrence of adverse perinatal outcomes and sociodemographic characteristics in our multivariable logistic regression model. There is a need for further studies to determine the pathomechanism of postpartum depression in this context.

Furthermore, it is possible that a rapid change in haemoglobin concentration (as in cases of postpartum haemorrhage) rather than a slow change (as with anaemia treatment with iron during pregnancy) is necessary for the occurrence of postpartum depression with respect to anaemia. Red blood cells are rapidly lost when haemorrhage occurs resulting in anaemia of sudden onset. This might cause an impairment in myelination within the brain and an alteration in monoamine metabolism leading to a fall in the levels of neurotransmitters like dopamine, serotonin, and norepinephrine with resultant mood disturbances [47]. In addition, hypotension might occur leading to cerebral hypoperfusion and ischemia which might in turn cause neuronal necrosis in parts of the hippocampus, a brain structure that has been implicated in depression [48]. 

Regarding the association between iron deficiency anaemia and postpartum depression, our finding is supported by that of a systematic review which found that iron deficiency occurring during pregnancy was not associated with an increased risk of postpartum depression, but does when iron deficiency occurred in the postpartum period, for instance, following postpartum haemorrhage [49]. This being a secondary analysis of data from a clinical trial, it would be worth exploring in future longitudinal studies, the association between anaemia and iron deficiency during the antenatal and postpartum period on the risk for postpartum depression at various time points and on a larger sample size.

The causes of postpartum depression are multifactorial. Apart from anaemia, other risk factors identified in an earlier study included lack of emotional and social support, family history of depression, and life stressors [50]. However, this study did not find an association between postpartum depression and sociodemographic factors like age, place of residence and income. Neither did it find an association with obstetric factors like gestational age, pregnancy complications apart from postpartum haemorrhage, or adverse foetal outcomes. Unfortunately, we could not adjust for social factors like lack of social support following childbirth, intimate partner violence and other negative life events because the clinical trial from which this study was extracted did not provide this information.

### Limitations

This study was a secondary data analysis and thus limited by available sample size which did not allow us to consider possible effect modification by postpartum haemorrhage or route of iron administration. We did not have a broad sample of patients’ characteristics because of the exclusion criteria applied in the clinical trial like exclusion of women with human immunodeficiency virus infection, autoimmune disorders, or severe asthma. In addition, we used only the English version of the EPDS tool to assess the women for depression. We excluded pregnant women with depression at enrolment (self-reported or EDPS > 10) to minimize confounding and bias because pre-existing depression is more likely to be due to life stressors which the clinical trial did not capture, rather than pregnancy-related events or anaemia. In addition, women with pre-existing depression might be on antidepressants which might alter their EPDS scores. The EPDS tool was used in this study to screen for depression in the antepartum and postpartum periods because it has been found to perform well in both periods in terms of factor model fit and reliability; however, it is important to note that the same score may not necessarily indicate the same level of depression at both time points [51]. 

Though generalizability of the study findings would be better if we had included states across regions of Nigeria, the IVON TRIAL participants in Kano State were excluded from this study because of language barrier and the lower literacy rate of the women which might cause some response bias with the use of the English version of the EPDS tool for assessing postpartum depression [52]. Regarding the best time to screen women for postpartum depression, there is a possibility that our earlier assessment of depression at two weeks postpartum did not give sufficient time for changes in mental health status to manifest compared to studies that screened at later time points. Kettunen et al. in a previous study found that 46% of women with postpartum depression were diagnosed by one-and-half weeks following delivery, 74% by four weeks, 84% by six weeks, and 98% by three months [53]. This suggests that the true prevalence of postpartum depression is likely higher than reported in our study, and this might have implications on the magnitude and direction of the association with antenatal improvement in anaemia or iron status. However, there is no consensus regarding the ideal time to screen for postpartum depression [54]. To the best of our knowledge, this is the first study evaluating the effect of an improvement in anaemia severity and iron deficiency on the risk for postpartum depression in sub-Saharan Africa.

## Conclusion

An improvement in anaemia severity during pregnancy was non-significantly associated with a lower risk of postpartum depression in this analysis. Improvement in iron deficiency was not associated with postpartum depression. Postpartum haemorrhage, which consequence is anaemia, increased the risk of postpartum depression. It would be of great benefit to determine the pathomechanism of postpartum depression following acute (e.g. postpartum haemorrhage) versus chronic (e.g. nutritional anaemia) events, to determine the best timepoint for assessment of postpartum depression, and to adjust for social factors and other life stressors in future research.

## Electronic supplementary material

Below is the link to the electronic supplementary material.

Supplementary Material 1

## Data Availability

The data for this research was obtained from the IVON trial dataset. The datasets analysed for this study can be made available on reasonable request from the lead/corresponding author of this manuscript.

## Abbreviations

95%CI: 95% confidence interval
EPDS: Edinburg Postnatal Depression Scale
g/dL: Grammes per decilitre
Hb: Haemoglobin
HICs: High-income countries
IVON: Intravenous versus oral iron for iron deficiency anaemia in pregnant Nigerian women
kg/m2: Kilogramme per square metre
LMICs: Low-middle-income countries
mL: Millilitre
ng/mL: Nanograms per millilitre
NHREC: National Health Research Ethics Committee
OR: Odds ratio
