# Supplementary material for: Is an improvement in anaemia and iron levels associated with the risk of early postpartum depression? A cohort study from Lagos, Nigeria
Source: BMC Public Health. 2025 Feb 28;25:808. doi: 10.1186/s12889-025-21942-x (PMC11869588; doi:10.1186/s12889-025-21942-x)
Supplement: Supplementary file 1 — Supplementary Material 1 [file 12889_2025_21942_MOESM1_ESM.pdf]

SUPPLEMENTARY FILES

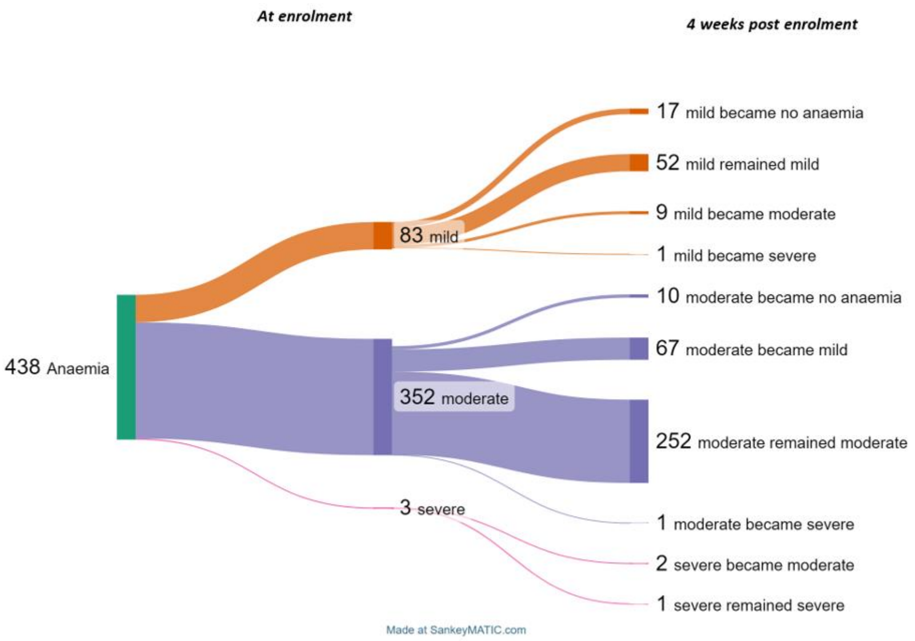

Of the participants who had enrolment haemoglobin concentration, 26 did not have haemoglobin concentration reported at four weeks post-enrolment and did not reflect in the Sankey flow diagram. The missingness was among women with mild anaemia at enrolment (n = 4) and those with moderate anaemia at enrolment (n = 22).

Figure SM1. Pattern of improvement in severity of anaemia from enrolment to four weeks post-enrolment

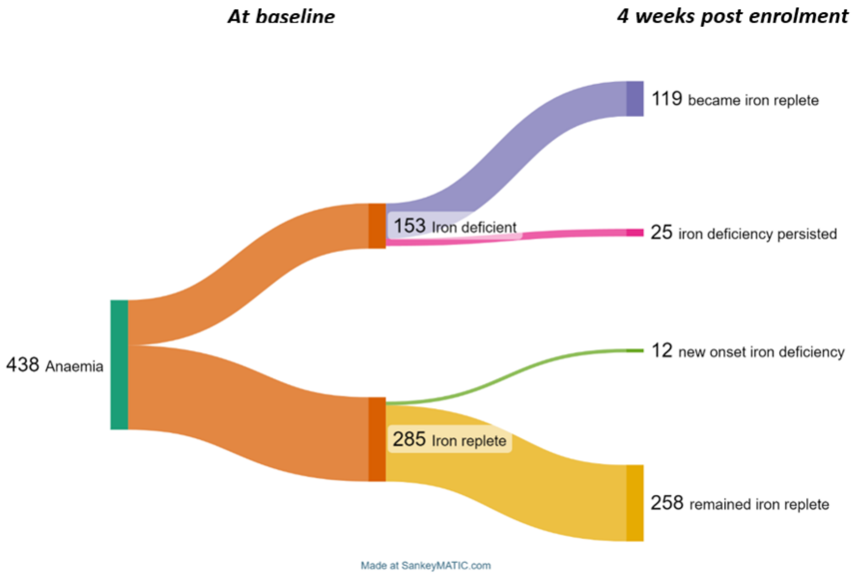

Twenty-four missing values for serum ferritin at four weeks post-enrolment, of which nine had iron deficiency at enrolment and fifteen were iron replete at enrolment.

Figure SM2. Pattern of improvement in iron levels from enrolment to four weeks post-enrolment

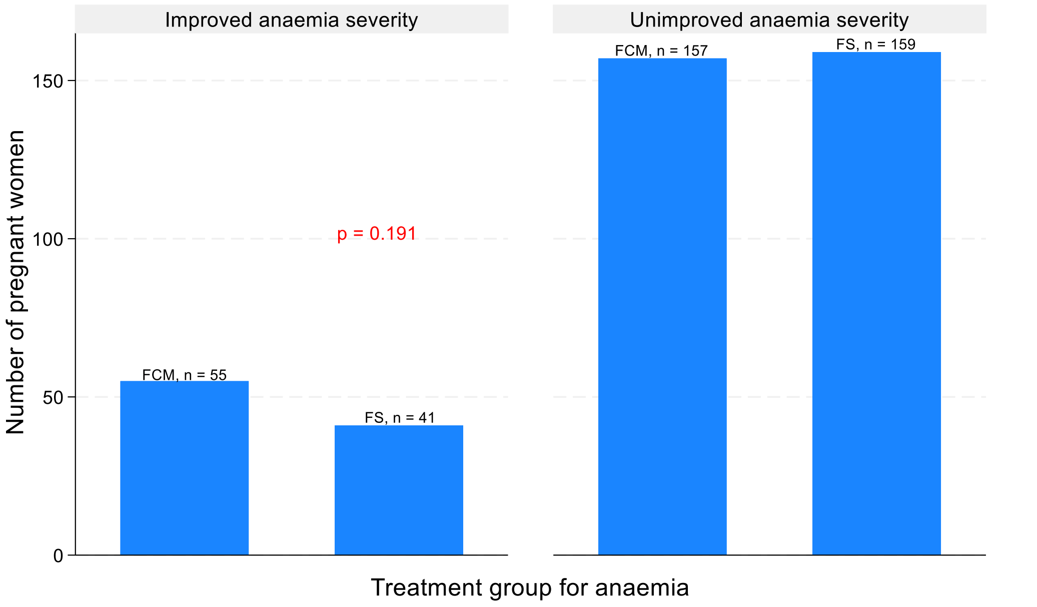

FCM – Intravenous ferric carboxymaltose, a single loading dose given at enrolment.
FS – Oral ferrous sulphate given three times a day all through pregnancy.

Figure SM3. Comparison of the improvement in the severity of anaemia by treatment group

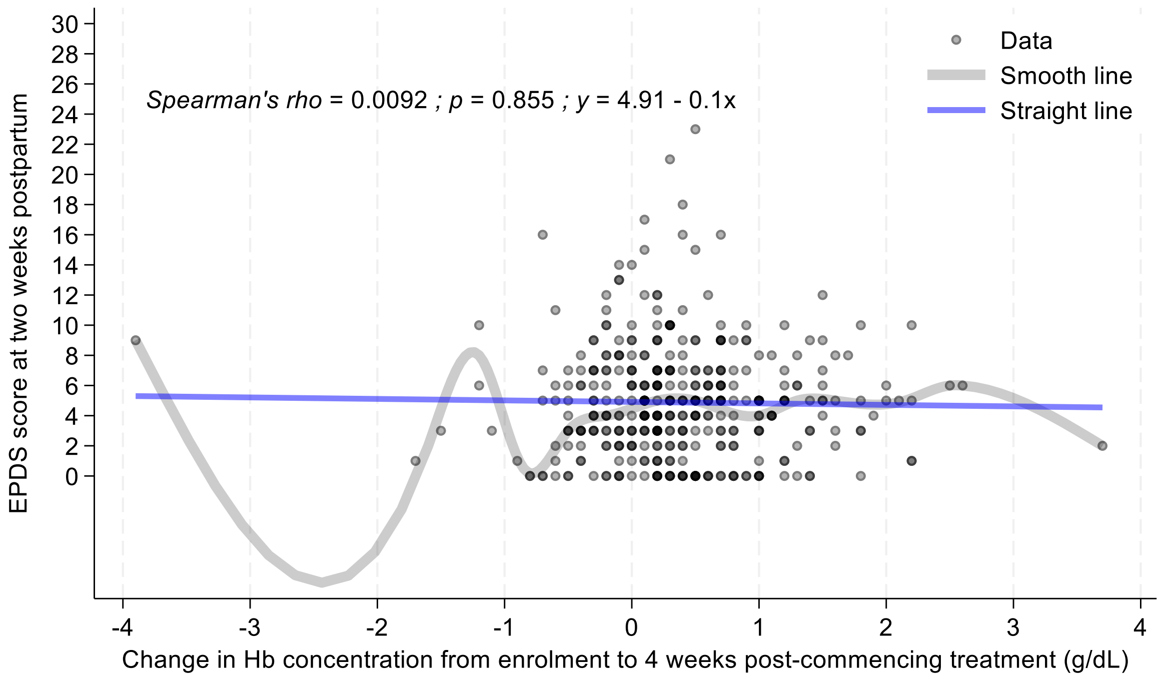

EPDS – Edinburg postpartum depression scale; Hb – haemoglobin; r is correlation coefficient; p – p-value; y = EPDS at two weeks postpartum and x = change in haemoglobin concentration from enrolment to four weeks post commencement of anaemia treatment.

Figure SM4. Association between change in haemoglobin concentration and EPDS score at two weeks postpartum

A.

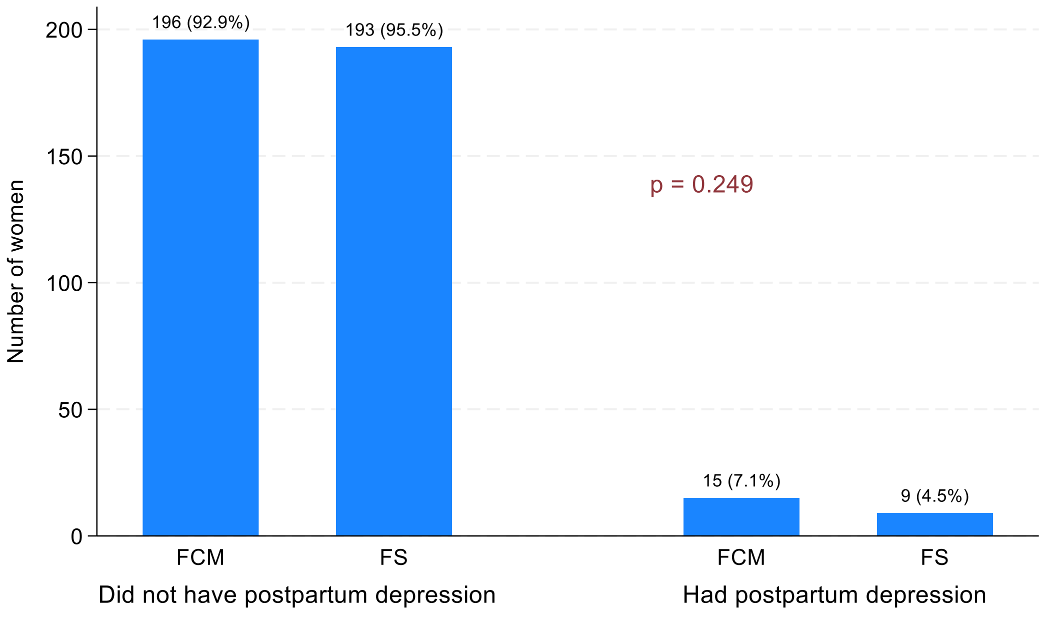

B.

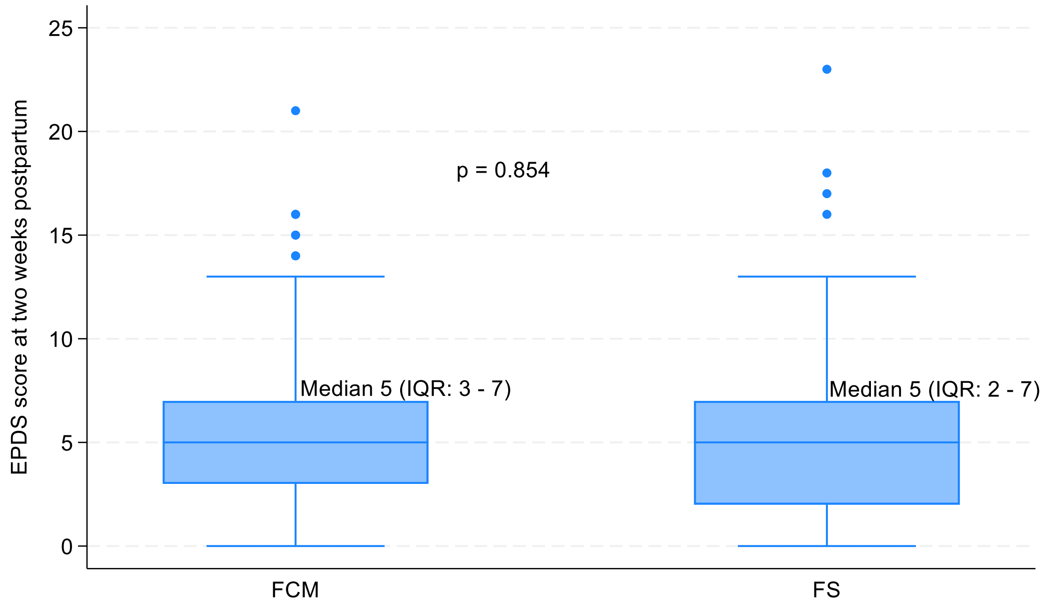

FCM – Intravenous ferric carboxymaltose, FS – Oral ferrous sulphate, IQR – Interquartile range.

Figure SM5. Comparison of prevalence of depression and median postpartum EPDS scores by study group.
